# Kinetic characterization of selective peroxisome-proliferator-activated receptor gamma modulators *in vitro*

**DOI:** 10.1186/cc11739

**Published:** 2012-11-14

**Authors:** T Knape, LK Eifler, A Heeg, L Kuchler, B Brüne, MJ Parnham, A von Knethen

**Affiliations:** 1 Fraunhofer IME Project Group - Translational Medicine and Pharmacology, Frankfurt, Germany; 2 Institute of Biochemistry I - Pathobiochemistry, Goethe-University Frankfurt, Germany

## Background

The ligand-activated transcription factor, peroxisome-proliferator-activated receptor gamma (PPARγ), has been shown to play an essential role in immunosuppression during sepsis. PPARγ is upregulated in T cells of septic patients, sensitizing these cells to PPARγ-dependent apoptosis and thus contributing to T-cell depletion [1,2]. In the polymicrobial cecum ligation and puncture (CLP) sepsis model in mice, both T-cell-specific gene knockout (Lck-Cre PPARγ^fl/fl^) and systemic pharmacological PPARγ antagonism by GW9662 improved survival [3]. Because GW9662 was only effective when applied 3 hours after CLP, we were interested to extend this time frame. For this reason we characterized the kinetics of SPPARγMs when administered before or in combination with the agonist thiazolidinedione, rosiglitazone.

## Methods

A PPARγ-dependent transactivation assay was used in HEK293T cells. It is based on the vector pFA-PPARγ-LBD-GAL4-DBD encoding the hybrid protein PPARγ-LBD-GAL4-DBD and the reporter vector pFR-Luc, carrying a GAL4-responsive element in front of the *Firefly *luciferase gene. These two vectors were co-transfected, in combination with a control vector encoding *Renilla *luciferase (pRL-CMV) to normalize *Firefly *luciferase activity for transfection efficiency. Following transfection, cells were incubated with the SPPARγMs F-MOC and MCC-555 and the PPARγ antagonist GW9662 for different times (2 to 48 hours) and at increasing doses (0.01 to 10 μM), with or without rosiglitazone (0.01 to 10 μM). Transactivation was analyzed using a 96-well plate format.

## Results

Rosiglitazone transactivated PPARγ in a time-dependent and dose-dependent manner, the response gradually increasing to a maximum at 48 hours with 10 μM. Low concentrations (0.01 to 0.1 μM) of SPPARγMs F-MOC and MCC-555 and the PPARγ antagonist GW9662 all exerted dose-independent antagonistic effects at an early incubation time point (2 hours). From 10 hours onwards, MCC-555 and GW9662, given alone, both exerted PPARγ agonistic effects, MCC-555 in parallel to responses to rosiglitazone, but GW9662 with characteristics of partial antagonism. F-MOC showed no dose-dependent effect at any concentration at later time points. Only GW9662 (1 to 10 μM) was able to inhibit rosiglitazone (0.1 to 1 μM)-induced PPARγ transactivation after 10 hours.

## Conclusion

Our kinetic analysis reveals clear differences in the modulatory characteristics of PPARγ inhibitors, with previously unreported early inhibitory effects and late agonistic or partial agonistic activity. New SPPARγMs with extended inhibitory activity may prove useful in the therapy of sepsis.
